# Supplementary material for: Perceptual cognitive abilities in young athletes: A gender comparison
Source: PLoS One. 2022 Aug 31;17(8):e0273607. doi: 10.1371/journal.pone.0273607 (PMC9432702; doi:10.1371/journal.pone.0273607)
Supplement: Attachment — Submitted filename: ResponseToReviewers.docx [file pone.0273607.s002.docx]

Dear Editor,

We are resubmitting our paper entitled “Perceptual cognitive abilities in young athletes: A gender comparison.” to you with the corrections suggested by you and the reviewer. Details can be found in our point-by-point response to the comments.

We hope that these corrections will satisfy your expectations.

Best Regards,

Isabelle Legault, PhD.

100, rue Duquet,

Sainte-Thérèse (Québec)

CANADA

J7E 3G6

PONE-D-21-37053

Perceptual cognitive abilities in young athletes: A gender comparison.

PLOS ONE 
 

1. Please ensure that your manuscript meets PLOS ONE's style requirements, including those for file naming. The PLOS ONE style templates can be found at  
https://journals.plos.org/plosone/s/file?id=wjVg/PLOSOne_formatting_sample_main_body.pdf and  
https://journals.plos.org/plosone/s/file?id=ba62/PLOSOne_formatting_sample_title_authors_affiliations.pdf

*Correction was made*

2. You indicated that you had ethical approval for your study. In your Methods section, please ensure you have also stated whether you obtained consent from parents or guardians of the minors included in the study or whether the research ethics committee or IRB specifically waived the need for their consent.

*The participants included in this study were allowed to give their consent. As specified in the Tri-Council Policy Statement: Ethical Conduct for Research Involving Humans – TCPS 2 (2018) in Canada, chapter 3, in minimal risk experiment, « Where a person has the capacity to understand this information, and the ability to act on it voluntarily, the decision to participate is generally seen as an expression of autonomy. ». Based on this definition and the nature of the present project, the ethic board has authorized* *our participants (aged 16 years old and over) to give their own consent.*

*The following sentence was added:*

*The ethic board has authorized our participant (aged 16 years old and over) to give their own consent, based on the low risk factor in this study.*

3. Please include captions for your Supporting Information files at the end of your manuscript, and update any in-text citations to match accordingly. Please see our Supporting Information guidelines for more information: http://journals.plos.org/plosone/s/supporting-information.

*Corrections were made*

**Comments to the Author** 
 
1. Is the manuscript technically sound, and do the data support the conclusions? 
 
The manuscript must describe a technically sound piece of scientific research with data that supports the conclusions. Experiments must have been conducted rigorously, with appropriate controls, replication, and sample sizes. The conclusions must be drawn appropriately based on the data presented.

Reviewer #1: Partly

2. Has the statistical analysis been performed appropriately and rigorously?

Reviewer #1: No

3. Have the authors made all data underlying the findings in their manuscript fully available? 
 
The PLOS Data policy requires authors to make all data underlying the findings described in their manuscript fully available without restriction, with rare exception (please refer to the Data Availability Statement in the manuscript PDF file). The data should be provided as part of the manuscript or its supporting information, or deposited to a public repository. For example, in addition to summary statistics, the data points behind means, medians and variance measures should be available. If there are restrictions on publicly sharing data—e.g. participant privacy or use of data from a third party—those must be specified.

Reviewer #1: Yes

4. Is the manuscript presented in an intelligible fashion and written in standard English? 
 
PLOS ONE does not copyedit accepted manuscripts, so the language in submitted articles must be clear, correct, and unambiguous. Any typographical or grammatical errors should be corrected at revision, so please note any specific errors here.

Reviewer #1: Yes

5. Review Comments to the Author 
 
Please use the space provided to explain your answers to the questions above. You may also include additional comments for the author, including concerns about dual publication, research ethics, or publication ethics. (Please upload your review as an attachment if it exceeds 20,000 characters)

Reviewer #1: The topic is interesting. However, the authors are recommended to reinforce the rationale linked to the gender differences within the introduction section. IMO, the fact that few studies have been conducted is not sufficient.

The Reviewer believe that a specific paragraph dedicated to the data analysis employed is needed.

*This section was added at the end of the Method section (data analysis):*

*The task measured speed thresholds obtained by each participant after 3 blocks of 20 trials (total of 60 trials per subject). At any given trial if the participant gets all targets correct the moving items speed up by 0.5 log units in the next trial. If the participant makes one or more mistakes in the recall, the next trial slows down by 0.5 log units. The speed threshold value of a given block consists of the mean of the speed for the last 4 reversals. First, the speed value used for statistical analysis for the entire session is the average of the 3 block thresholds obtained for each of the participants. We are going to compare, by a statistical analysis (split-plot ANOVA) whether differences remain between groups and evaluated with Post hoc Bonferonni correction if significant interactions are obtained.*

*Finally, we predict a different improvement (speed thresholds) within groups between block 1 and block 3. To perform this analysis, we will subtract the results obtained in the 3rd trial from those obtained in the first and we will perform a t-test on these results. A significant difference will tell us that there is a difference in the speed of learning in our participants between trials 1 and 3.*

The results section was modified:

*IBM SPSS statistics version 24 (SPSS, Chicago, Illinois) was used for all statistical analysis, with alpha set at p ≤ 0.05. A split-plot ANOVA on speed thresholds comparing groups revealed a significant group effect, F (3,80) = 6.789 p < 0.01, η^2^ = 0.698. Subsequently, Post hoc comparisons using the Bonferonni correction indicated that the mean score for the male athlete group (M = 1.73, SD =0.48) was significantly different than female athletes and nonathlete males and females, see Table 1.*

*As observed in Fig 2, male athletes reached higher speed thresholds than other groups. Furthermore, a paired t-test was performed on normalized data to observe the progression of the participants' speed thresholds between the first and last trials. The NT task is sensitive to repetition, and participants improve their performance significantly trial after the trial [1-3]. Normalized data showed differences between female athletes and other groups, leading to a more pronounced performance progression in the female athlete group (Table 2).*

*Table 1: Post hoc comparisons using the Bonferonni correction*

| *(I)groups J (groups)* | | *Mean difference (I-J)* | *Std. Error* | *Sig.* |
| --- | --- | --- | --- | --- |
|  |  |  |  |  |
| *AF* | *AM* | *-,43373^*^* | *0.12462* | *0.005* |
|  | *NAF* | *0.07179* | *0.12609* | *1.000* |
|  | *NAM* | *-0.05516* | *0.12462* | *1.000* |
| ***AM*** | ***AF*** | ***,43373^*^*** | ***0.12462*** | ***0.005*** |
|  | ***NAF*** | ***,50552^*^*** | ***0.12297*** | ***0.001*** |
|  | ***NAM*** | ***,37857^*^*** | ***0.12146*** | ***0.015*** |
| *NAF* | *AF* | *-0.07179* | *0.12609* | *1.000* |
|  | *AM* | *-,50552^*^* | *0.12297* | *0.001* |
|  | *NAM* | *-0.12695* | *0.12297* | *1.000* |
| *NAM* | *AF* | *0.05516* | *0.12462* | *1.000* |
|  | *AM* | *-,37857^*^* | *0.12146* | *0.015* |
|  | *NAF* | *0.12695* | *0.12297* | *1.000* |

*A : athletes / NA : non-athletes / M : male / F : female*

*Table 2: Paired t-test results for normalized data.*

| *Groups* | *Normalized data* |
| --- | --- |
| *A/M-A/F* | ***t (1,18) = 2.152, p = 0.045*** |
| *A/F – NA/F* | ***t (1,18) = -2.594, p=0.018*** |
| *A/M-NA/M* | *t (1,19) = -0.211, p = 0.943* |
| *NA/F-NA/M* | *t (1,19) = -0.72, p= 0.835* |

*A : athletes / NA : non-athletes / M : male / F : female*

 
Here below some specific comments: 
 
Line 82: please reinforce the rationale of the study, explaining why it should important including a gender comparison.

*The following paragraphs were modified:*

*Although the level of the athlete is associated with better perceptual cognitive abilities, where neural changes are observed in functions required during the training such as attention (divided / sustained or selective)[4],it remains unclear whether there is a gender difference in both male and female athletes’ cognitive abilities.* Voss et al. (2009) meta-analytic review suggested that male athletes observed a greater cognitive benefit than female athletes compared to the non-athlete control group but suggested that more studies recruiting both male and female are needed to permit more accurate data interpretation [5].

*Moreover, there is a lack of literature on gender differences in the evaluation of perceptual cognitive ability tasks. Several studies show gender differences across life span on spatial cognition [6-9]. More relevant to the present study, Roudaia and Faubert [29] supports a significant gender difference in attentional tracking. Their results showed gender differences in tracking performance for younger and older adults, where males obtained higher tracking speed, which indicates a higher temporal resolution of attention. Furthermore, researchers have found a lower reaction time in females, which can be explained by their lower ability in attentional function [37-40] and revealed that females are more negatively influenced by distractors [41, 42].*

line 95: the authors are invited to provide their study hypothesis in line with the logical flow of the introduction.

*Correction was made:*

*The purpose of the present study was therefore to establish whether gender differences are observed for tracking multiple moving objects in athlete and non-athlete populations.*

 
line 99: please justify the sample size included

*Our sample size for 2 groups with a Power 1- β = .8 and Type I error rate, α = 0.05, was estimated at 63 participants. We decided to use 80 participants to ensure enough power.*

*The following was added:*

*Our sample size for 2 groups with a Power 1- β = .8 and Type I error rate, α = 0.05, was estimated at 63 participants.*

Moreover, report more details about participants?

The following sentence was added:

*Participants were recruited from the college population, and they were, for the majority, in a pre-university program.*

Anthropometric data?

*As our task was a visual perception task, the anthropometric data (such as weight and size) was not collected as this is expected to have no relevant impact on our measures.*

*We have now included viewing distance, screen size and degrees of visual angle of the stimulus used in the study in the procedure section.*

The following sentence was added:

*The participants were seated at 30 cm from the screen and asked to stare at the fixation point, located straight ahead. Stimuli consisted of height spheres projected into a virtual cube measuring 324 cm^2^. The anterior side of the cube measured 33.4 ° of visual angle*

A specific section for data analysis is needed. Please provide it.

*Correction was made, see the first comment*

 
line 171: redundant. Remove it

*The sentence was removed* 

Provide also some practical information to the readers dealing with young athletes.

*Added in the text:*

*As mentioned above, Athletic training creates brain modifications resulting in benefits in attention processing, decision making, etc. [4]. On the other hand, these attentional processes can be trained and can potentially lead to better performance on the field [2]. By increasing attention processes, athletes can potentially become more performant. Alternatively, athletic training can benefit the plasticity for attention mechanisms. As was demonstrated by Faubert (2013) sport expertise has a dramatic impact on attentional capacities for processing dynamic scenes as measured by the 3D-MOT task [10]. Therefore, it may be beneficial for female athletes to increase their participation in sports activities. However, it remains to be determined at what level the gap seen between males and females in attention mechanisms responsible for the 3D-MOT can be closed by engaging in competitive sports.*
